# Regulating human genomic research in Africa: why a human rights approach is a more promising conceptual framework than genomic sovereignty

**DOI:** 10.3389/fgene.2023.1208606

**Published:** 2023-06-30

**Authors:** Faith Kabata, Donrich Thaldar

**Affiliations:** ^1^ School of Law, University of KwaZulu-Natal, Durban, South Africa; ^2^ Petrie-Flom Center for Health Law Policy, Biotechnology and Bioethics, Harvard Law School, Cambridge, MA, United States

**Keywords:** human genomics, genomic sovereignty, human rights, regulation, right to science, Africa

## Abstract

This article revisits the debate on the regulation of human genomic research, with a focus on Africa. The article comprehensively examines the concept of genomic sovereignty, which was invoked mainly in the global South as a conceptual framework for state regulation of human genomic research. It demonstrates that genomic sovereignty has no utility value in human genomic research as it violates the rights of individuals and researchers. By analysing Mexico’s regulatory approach based on genomic sovereignty and a divergent regulatory approach, viz Finland’s human genomic research framework, we show that a human rights approach is more promising as it aligns with the state obligations under the right of everyone to participate in and benefit from scientific progress and its applications in international human rights law. We conclude by recommending that African states should anchor regulation of human genomic research on a human rights framework based on the right to science.

## 1 Introduction

Is state regulation of access to and use of genomic material the appropriate governance framework for human genomic research? This article offers insights in the debate on regulation of human genomic research. It does this by examining the concept of genomic sovereignty to enquire if the concept has utility value in human genomic research, particularly in relation to Africa. The short answer is “no.” The longer answer, and specifically why the concept has no utility value, however, leads to important questions on: the concept, its underpinnings, assumptions and weaknesses; other divergent state approaches in regulation of human genomic research; and questions on where state approaches to the regulation of human genomic research should focus their attention.

To situate the discussion, the article revisits deliberations of the UNESCO International Bioethics Committee during the drafting of the Universal Declaration on the Human Genome and Human Rights. A member of the Committee stated (UNESCO International Bioethics Committee, 1995):

“We are now proposing to include the human genome in the common heritage of humanity. Legally speaking, this would be a historic and revolutionary measure, fraught with implications and attended by many consequences that would be beneficial to humanity.”

Illustratively, the Universal Declaration on the Human Genome and Human Rights refers to the genome as “heritage of humankind,” thus seemingly opening the possibility of the common heritage framework of governance (UNESCO, 1997). In response to this, a number of countries in the global South invoked sovereign claims over the genetic material of their citizens as a perceived way of protecting it from foreign exploitation by the global North. These claims were embedded in the concept of genomic sovereignty, which assumed a political and scientific agenda and was incorporated into domestic legislation or guidelines in some countries. Importantly, the concept put into sharp focus the role of the state in regulating access to and use of genomic resources, an issue that remains relevant for Africa.

The article flows as follows: Part 2 draws on an array of genomic sovereignty literature to map out its argumentative logic, and identify its themes and conceptual weaknesses. Part 3 examines Africa’s academic engagement with genomic sovereignty, the conceptual underpinnings, and critically assesses the concept. Part 4 reviews Finland’s approach to human genomic research, its philosophical underpinnings, and conducts a comparative analysis of both the Mexican and Finnish approaches. Part 5 addresses the question of where approaches to regulation of human genomic research should turn by exploring state obligations in the right to science and specifically in human genomic research. Part 6 summarises the article by offering concluding thoughts.

The article uses the terms developing and developed countries and global South and global North interchangeably, as they are used in the literature referenced. The article also acknowledges that the definition of genomic sovereignty in the literature relied on the terms *control* and *ownership* of genetic resources interchangeably, which is legally problematic.

## 2 Unearthing genomic sovereignty

In the context of global research, genomic sovereignty has been referred to as the ability of a nation, people or state to own and regulate access to and use samples, data and knowledge on human genes (Slabbert and Pepper, 2010).

The term was coined by Mexican scientists, politicians and policy makers as a biopolitical concept describing political sovereignty in genome mapping and was aimed at protecting national genomics in Mexico (Marìn-Schwartz, 2011). The concept was typified by establishment of the National Institute of Genomic Medicine (INMEGEN) in 2004; the mapping of the “Mexican genome” by the INMEGEN between 2004 and 2009; and the framing of the policy agenda into legislation to protect Mexico’s “genomic sovereignty” in 2008 (Vasquez and García-Deister, 2019). The legislation referred to as Mexico’s policy on genomic sovereignty instituted amendments in the General Health Law (Marìn-Schwartz, 2011).

The law was designed to regulate everything in the human genome in Mexico. Accordingly, it restricted the movement of biological samples outside Mexico for population genomics studies without express authority from the Secretary of Health and attached penalties of 15 years imprisonment and imposition of fines for unauthorised movement (Marìn-Schwartz, 2011). In addition, it implicitly addressed intellectual property in two ways: first, if genetic material was taken outside Mexico without authorisation no intellectual property claims would be recognised; and second, if there were no benefits to Mexico, intellectual property claims would not be recognised (Marìn-Schwartz, 2011). Significantly, according to the proponents, the law was not meant to impede research, but rather to spur international research collaboration through a permit system (Marìn-Schwartz, 2011). The import of the law was that the state sought to control access to organ, tissue or human components of living or dead persons. The most incisive criticism of the law on genomic sovereignty is that it was anchored on the uniqueness of the Mexican genome, which it assumed could be uniquely identified and policed internationally (Marìn-Schwartz, 2011). This discussion is fully taken up in part 2.2.

Beyond Mexico, the concept of genomic sovereignty also had policy underpinnings in India and Thailand and it generated some academic interest in South Africa. In India, genomic sovereignty was similarly conceived as a policy and scientific regime to prevent unapproved movement of genomic material and data outside India based on the need to secure state investment in genomic research and to ensure that local researchers benefit from their discoveries (Sèguin et al., 2008). The basic premise of India’s genomic sovereignty was its population, which was viewed as a resource because of its large size and its uniqueness given the practice of multi-generational endogamy and the existence of proper genealogical records (Sèguin et al., 2008). The genomic sovereignty agenda was thus typified by review of Guidelines for Exchange of Human Biological Material for Biomedical Research Purposes, making it mandatory to obtain government permission to export human biological material (Hardy, 2011).

In Thailand, genomic sovereignty, though not as explicit as in Mexico and India, was similarly conceptualised as a policy agenda to protect the “Thailand genome,” specifically DNA samples, from export (Sèguin et al., 2008). While legislative action was not undertaken, there was debate among researchers on the need to strengthen existing guidelines into law in order to limit export of Thai DNA samples (Sèguin et al., 2008).

### 2.1 Conceptual underpinnings of genomic sovereignty

From the foregoing, the concept of genomic sovereignty is rooted in post-colonial discourses of dispossession. The concept thus did not arise unexpectedly but should be regarded as an extension of the continuing North/South tension over dispossession and foreign exploitation of national resources. Illustratively, Marìn-Schwartz (2011) indicates that while the concept is traced to Mexico, it appears to have been adapted from arguments in international fora such as UNESCO in relation to indigenous peoples’ genetic heritage and tribal knowledge. While *sovereignty* has always been invoked by states, groups or peoples claiming control of natural resources, in this context it was bundled together with the catchword *genomic* to refer to the new national resource, and hence genomic sovereignty.

The argumentative logic that underpins genomic sovereignty is that in the genomic era, national population genomes can be mapped and controlled. Proponents of the concept thus conflate population with territory and as states ordinarily exercise control over national resources in their territory, then they argue that states can assert control over the population genome in their territory as a national resource (Marìn-Schwartz, 2011). This logic is premised on the idea that the human genome has commercial and symbolic value, and hence commercially as a source of economic revenue and symbolically as a source of national pride and identity (De Vries and Pepper, 2012).

Drawing from the logic of national resources and the history of colonial dispossession, genomic sovereignty is grounded in the inter-linked themes of: economic gain, national heritage and patrimony (Hardy, 2011). The economic gain argument is anchored on bio-value to be derived from the genomic revolution (Vasquez and García-Deister, 2019). For instance, Hardy (2011) captures this sentiment in relation to India: “If oil in Alaska can be shared by everybody, (the) Indian genome of India can be shared by everybody. But the fact is that (the) oil of Alaska is not shared by everybody”. Strikingly, this comparison of the Indian genome to oil depicts the view of the genome as a national resource and the economic value attached to it, and hence the need to protect it from foreign exploitation.

Similarly, in Mexico, Siqueiros-Garcìa et al. (2013) point out that debate on the genomic sovereignty law drew from past foreign exploitation of petroleum, archaeological resources and biodiversity. Genomic sovereignty was thus to ensure that the Mexican genome was analysed by Mexicans and for Mexicans.

Related to the economic argument is the theme of national heritage, which is premised on the symbolic and cultural value of the human genome. The theme is anchored on the idea that national populations are biologically distinct from other populations, and hence national populations are branded as biological units (Marin-Schwartz and Mendez, 2012). National heritage is thus linked to self-determination and national building for developing countries, with the genetic make-up of the population viewed as a national resource whose exploitation is a nation-building project to deliver specific health outcomes for the national population and ensure participation in the global knowledge-based economy (Benjamin, 2009; Vasquez and García-Deister, 2019). The utility of the nation heritage theme achieved public support for genomic research for two reasons. First, to justify heavy financial investment in genomics research in light of more immediate public health concerns (Benjamin, 2009; Vasquez and García-Deister, 2018); and, second, to secure public uptake which is critical as a source of biological samples (Hardy, 2011).

Finally, the theme of patrimony, which closely mirrors national heritage, suggests protection from bio-exploitation. The central idea is that the national genome can be defined, separated from other populations, and the state can assert sovereignty over the national genome based on patrimonial doctrines (Marin-Schwartz and Mendez, 2012). In this sense, genomic sovereignty meant the duty to protect the genome of populations based on the notion of property. Illustratively, Mexico’s genomic sovereignty law described the Mexican genome as a public good, a sovereign resource, which implied that the Mexican government could police and control it internationally (Marìn-Schwartz, 2011). In addition, the patrimony theme alludes to protective control, captured with the expression “genomics by Mexicans, in Mexico and for Mexicans” (Vasquez and García-Deister, 2018).

### 2.2 Gaps in the concept of genomic sovereignty

The concept of genomic sovereignty as discussed above provokes several questions: Is the claim of a national genome that can be uniquely identified and over which states can assert sovereignty feasible in genomic research? What exactly does genomic sovereignty relate to? Is it biological samples, data, or both? Are genomic sovereignty laws and regulations enforceable?

The Human Genome Project determined that 99.9% of human DNA is similar, with only a 0.1% variation. This unique pattern of variation across populations is at the heart of genomic research. This leads to the question of how the population of interest should be constituted, and how should the population with the unique pattern of variation be mapped? Genomic sovereignty is built on the assumption that the population of the state–based on shared national identity–constitutes a unique genetic mixture, distinct from other nations, which the state then seeks to assert control over (Benjamin, 2009).

A good starting point for analysis on this is to adopt Marin-Schwartz’s and Mendez’s observation in relation to Mexico (Marin-Schwartz and Mendez, 2012):

“It is technically feasible to speak of sovereignty when we speak of the individual genome, which is unique; but to speak of sovereignty over the genome of a whole population is pretty difficult. We cannot speak of a unique Mexican make-up, when we are talking of shifting percentages of DNA fragments which are shared by humanity and various populations across the world.”

The picture that emerges from Marin-Schwartz’s observation is that a nation state’s genetic make-up cannot be mapped, defined and separated from that of other world populations. This point finds support in De Vries and Pepper (2012) who also note that, scientifically, genomic information for groups or populations in a country is not unique or distinct. The non-existence of a unique nation state’s genetic make-up raises questions that are at the core of the concept of genomic sovereignty.

Turning to the related question of whether the state can assert sovereign control over the genetic make-up of its population, Marìn-Schwartz (2011) observes that the sovereignty claims made by the state as policing genetic information, revoking intellectual property rights, and surveillance over Mexican samples were impractical. First, there is the issue of diasporic populations as law based on sovereignty articulations is territorial, while in the context of genomics, populations are fluid and are found outside state boundaries. Secondly, there is the nature of genomic research in which genetic information flows in the international information networks as part of large-scale transnational data sets which do not conform to the territory of the nation state. Thirdly, the individual and collective property rights dimension that arise make property and patrimonial doctrines unsuited to regulate population genomics (Marìn-Schwartz, 2011).

In the same strand of arguments, Vasquez and García-Deister (2019) allude to the impracticability of sovereignty claims in the context of genetic information flow by pointing out that, despite the genomic sovereignty law, Mexican samples and DNA were analysed and reduced into cloud data which flowed internationally without being confined to the political boundaries of the state.

On the question of whether genomic sovereignty relates to biological samples, data, or both, the text of Mexico’s genomic sovereignty law was silent on data. However, Marìn-Schwartz (2011) asserts that the proponents of genomic sovereignty viewed data as also protected from export without government approval. He observes that the proponents of Mexico’s genomic sovereignty variously indicated that “… what is being protected is knowledge about genes …”. On the contrary, Siqueiros-Garcìa et al. (2013) are categorical in their assertion that data is beyond the reach of genomic sovereignty. In addition, Rojas-Martìnez (2015) states that data is out of the scope of reach of Mexico’s genomic sovereignty law. Similarly, in India, the Guidelines for the Exchange of Human Biological Material for Research Purposes, which are at the heart of the genomic sovereignty agenda, control the export of human biological material (Ministry of Health and Family Welfare [India], 1997), while in Thailand, the regulations control export of human DNA samples (Sèguin et al., 2008).

It should be accepted that biological samples are the locus of data and information. Even then, two strands of argument defeat genomic sovereignty claims over control of genomic data. First, prevailing state practice among research communities is to openly share genomic data (Contreras and Knoppers, 2018). Marìn-Schwartz (2011) alludes to this in his analysis on the impracticability of Mexico’s genomic sovereignty policy, noting that Mexico is part of the international open access network to which it contributes. Secondly, and related to this, genomic research favours large data sets which circulate in the international system without being tied to nation states, and thus national data becomes less valuable. For example, Vasquez and García-Deister (2019) in their evaluation of Mexico’s genomic sovereignty find that after publication of data on the Mexican genome mapping, international collaborations shifted the research to large data sets resulting in de-centring of the Mexican genome in favour of Latino genomic data.

Concerning enforcement of genomic sovereignty laws, it focuses on the question of whether the concept is workable. This discussion on the practicality of the concept is fully taken up in part 3.3. Instructively, in the three countries, the laws, regulations and guidelines that underpinned the genomic sovereignty agenda were not enforced. In Mexico, a number of shortcomings in the law informed this outcome. First, the non-existence of the unique Mexican genome, which questions what was to be protected as Mexican “uniqueness” (Marìn-Schwartz, 2011). Secondly, Siqueiros-Garcìa et al. (2013) point to lack of institutional and administrative procedures to implement the law. This view is supported by Marìn-Schwartz (2011) who, based on participant observation, points out that even if the law implicitly purported to control intellectual property in Mexican genomic research, there were no mechanisms put in place. Thirdly, there is the design of the law, in that it sought to regulate population genomics, which is fluid, while articulations of state sovereignty traditionally regulate fixed objects (Marìn-Schwartz, 2011). Similarly, in India, lack of administrative and institutional support hindered the enforcement of guidelines protecting exportation of human samples without government approval (Hardy, 2011).

## 3 African academics’ engagement with genomic sovereignty

### 3.1 Overview

The literature on genomic sovereignty by African academics is dominated by the views of a South African academic, Pepper, and his collaborators. Accordingly, this section explores in a chronological fashion the development of the opinions of Pepper and his various collaborators on the topic.

Spurred by developments in Mexico, the discourse on genomic sovereignty gained international traction around 2010 (Marìn-Schwartz, 2011). At this time, Pepper published his first article on the topic. Slabbert and Pepper (2010) titled their article “A room of their own: Legal *lacunae* regarding genomic sovereignty in South Africa” and captured prevailing sentiments in the global South on the need for these countries to protect their genomic resources from exploitation by the global North. They linked genomic sovereignty to access to and benefit sharing in genomic research, particularly when genetic material originated from South Africa. They defined genomic sovereignty as “the capacity of a people, a country or a nation to own, to control both access to and use of samples, data and knowledge concerning or emanating from genomic material” which aptly captured the protection from foreign exploitation discourse. In this regard, they highlighted the need for laws to regulate individual data and the export of biological samples from South Africa.

Two years later, De Vries and Pepper (2012), in an article titled “Genomic sovereignty and the African promise: Mining the African genome for the benefit of Africa” explored whether the concept can protect genomic resources in the global South from exploitation by the global North. Pointedly, by 2012, Mexico’s genomic sovereignty policy had failed. In this article, De Vries and Pepper took a decidedly more critical view of genomic sovereignty. The authors acknowledged the appeal of the concept of genomic sovereignty in the African context, but pointed out its conceptual limitations. They identified the limitations as: lack of clarity on whom the final authority on access to and use of genomic material rests and the role of the individual donor; inability of states to represent the interests of the populations within their borders equally, including the contestations by indigenous peoples on representation of their interests; existence of ethnic groups across geographical state boundaries; the assumption that the population of a state is a unique biological unit; and the transnational nature of genomic data. Based on these limitations, the authors argued that genomic sovereignty is inadequate on its own to resolve the problems of inequality and unfair distribution of benefits in African genomic research.

Furthermore, in 2017, while discussing the exporting of DNA, Pepper alluded to the need to strike a balance between prevention of exploitation and promotion of innovation. In the discussion on ownership of DNA, he mentioned the concept of genomic sovereignty as referring to the “need to regulate ownership of human genetic resources” (Pepper, 2017).

Beyond academic research, policy proposals that have alluded to genomic sovereignty in South Africa have similarly been associated with Pepper’s involvement in the development of such proposals. For example, in 2011, the National Biotechnology Advisory Committee, of which Pepper was a member, published a statement on genomic sovereignty calling for public debate in South Africa on regulation and monitoring of human genomic material (National Biotechnology Advisory Council [South Africa], 2011). Most recently, in 2018, the Academy of Science of South Africa (ASSAf) published a report by a consensus study group, led by Pepper, on human genetics and genomics in South Africa. This report explicitly advanced the notion of an individual’s DNA and genomic data as being “natural resources,” to be managed by the state similarly to water or mineral resources (Academy of Science of South Africa (ASSAf), 2018).

### 3.2 Conceptual underpinnings of genomic sovereignty by African academics

What then underpins the concept of genomic sovereignty as espoused by the African academics discussed above? A textual analysis of Pepper and his research collaborators’ academic work yields repetition of the concepts: inequality; exploitation in export of samples; ownership of genomic resources; and unfair distribution of benefits. Based on this, it is therefore plausible to argue that the conceptual underpinnings of genomic sovereignty by African academics are rooted in the history of colonial dispossession, as in Mexico. De Vries and Pepper (2012) allude to this by linking genomic sovereignty to concerns of “revival of colonialist relations between Africa and the western world” in genomic research. In the same strand of argument, these authors state that genomic sovereignty offers a “conceptual framework” for genomic research by regulating ownership of genomic material and samples (De Vries and Pepper, 2012).

Pepper and fellow researchers couch the concerns on dispossession as inequality between local and international researchers, arguing that local researchers do not benefit from international research collaborations. According to these authors, exploitation in the export of samples is comparable to exploitation of natural resources such as oil and minerals, and can be addressed if the concept of genomic sovereignty is enshrined into law.

De Vries and Pepper (2012), while discussing the conceptual limitations of genomic sovereignty, also point out that the concept of genomic sovereignty is inadequate on its own in achieving equity and justice in genomic research. Rather, they highlight the need to develop governance tools to ensure fair distribution of benefits among researchers and populations. Pepper’s work on the export of DNA in South Africa revisits genomic sovereignty in the context of regulation of ownership of human genetic samples to guard against exploitation in research, and points out that it remains debated (Pepper, 2017).

### 3.3 Critical assessment of genomic sovereignty

A significant weakness in the literature discussed above is a failure to consider human rights. Most prominently, as pointed out by Thaldar et al. (2019), positing an individual’s genomic material and data as “natural resources”—similar to minerals and water—can be deemed offensive to individual dignity. In addition, the literature discussed above fails to recognise and deal with the right to freedom of scientific research, which is protected as a fundamental human right in some African countries, such as South Africa, Kenya, Morocco and Zimbabwe (Thaldar and Steytler, 2021). This failure to consider human rights raises the question of whether a legislative or policy move towards genomic sovereignty would withstand constitutional scrutiny.

Moreover, in the case of genomic data, one also needs to consider informational privacy rights. Various African countries have enacted data protection legislation. These include some of Africa’s most populous countries, such as Kenya, Nigeria, South Africa and Tanzania. Genomic data will typically fall within the scope of these statutes. Also, it is unlikely that genomic data can be de-identified or anonymised (depending on the terminology used in the specific jurisdiction) in order to escape the applicability of these statutes. The informational privacy rights protected in these statutes are therefore likely to apply to genomic data. How does this effect genomic sovereignty? Informational privacy rights belong to *individuals*, and aim to protect *individual* privacy interests. This stands in contrast with genomic sovereignty, which aims to promote *collective* ethnic group interests or state interests. It is not clear from the literature discussed above how proponents of genomic sovereignty propose to solve this philosophical dilemma.

Furthermore, to the extent that genomic sovereignty is understood as entailing *ownership* of genomic material and data by the state, such version of genomic sovereignty would amount to the nationalisation—and *expropriation*—of property that is currently *privately* owned. For example, in South Africa, genomic material is currently owned by the research institution to which such material is donated by a research participant (Thaldar and Shozi, 2022), and genomic data, once sequenced and saved as a digital object, can also be privately owned—likely by the research institution that performed the sequencing (Thaldar et al., 2022). Therefore, if *private* ownership of genomic material and data is replaced with *state* ownership, it means that such genomic material and data are expropriated, which in turn triggers legal protections of property rights. At the very least, the state would have to offer financial compensation to the private owners. The property law dimension of genomic material and data is a legal fact that cannot be ignored or wished away.

## 4 Finland’s state approach to human genomic research

### 4.1 Finland’s human genomic research infrastructure

This section discusses Finland’s human genomic research framework as follows: biobanking infrastructure; framework for availability and utilisation of genomic data, including the National Genome Strategy; proposed genome centre and Genome Act; and the Finngen project.

Finland’s framework on human genomic research traces back to 2006 when the Ministry of Social Affairs and Health established a working group to develop a law to regulate biobank operations in Finland (Salokannel et al., 2019). The Finnish Biobank Act entered into force in September 2013 and sets its objectives as supporting research that uses human biological samples, promoting openness in the use of the samples, and securing protection of privacy and self-determination when processing the samples. It regulates all types of biobanks and biological samples and information associated with the samples. The scope of the Act covers: establishment and operation of biobanks; collection of biobank samples and information attached to the samples; storage and processing of samples; rights of registered individuals to protect their privacy; and registers for biobanking (Ministry of Social Affairs and Health [Finland], 2013). The biobanks own the samples and are regarded as common resources, to which researchers have access. The Act allows broad consent for future research and secondary use of samples and the linking of personal data with the biobank information (Ministry of Social Affairs and Health [Finland], 2013). As of 2022, there are 11 registered biobanks in Finland, of which ten are public and one is private. All biobanks must obtain a licence from the Finnish Medicines Agency, Fimea (Ministry of Social Affairs and Health [Finland], 2013).

In 2015, Finland launched the National Genome Strategy which sets measures for incorporation of genomic data in the Finnish healthcare system by 2020 (Ministry of Social Affairs and Health [Finland], 2015). The Strategy is primarily focused on data collection and utilisation and the establishment of a single entity for management of genomic data. A key feature of the Strategy is the establishment of a new public authority, the genome centre, to promote responsible and equal use of genomic data. The genome centre will be mandated to: set up a national reference database of genomes; operate as a service point for research agreements, contracts and commercialisation; promote ethical practices in the use of genomic data by planning and implementing consents; facilitate networking and international collaboration; and initiate and stimulate public debate on utilisation of genomic data. The genome centre is envisioned as a permanent entity established through legislation, the Genome Act. The main objective of the Genome Act is to facilitate responsible, equal and secure processing of genomic data for the benefit of citizens (Ministry of Social Affairs and Health [Finland], 2015). As of 2022, the Genome Act was still being drafted.

In 2017, Finland launched the Finngen study, a national public–private research project to collect and analyse genome and health data from 500,000 participants of the Finnish biobanks by 2023. The study is a partnership between universities, hospitals, biobanks, the National Institute for Health and Welfare, international pharmaceutical companies and the Finns. As of December 2022, the total number of participants was 342,499 (FINNGEN, 2017). The aim of Finngen is to build a data resource that combines nationwide biobank data, national healthcare data, and genome data.

Finland’s strengths which have enabled the establishment of the above discussed research infrastructure are: high standard and universal healthcare; uniform treatment practices; national health registers; history of genetic research; and a population that is willing to participate in genomic research (Ministry of Social Affairs and Health [Finland], 2015).

### 4.2 Philosophical underpinnings of Finland’s human genomic research framework

Finland’s human genomic research framework is founded on individual (donor) sovereignty. Notably, Finland operates a welfare public healthcare system for all residents. Writing on data donation and exercise of sovereignty, Hummel et al. (2019) argue that individual data sovereignty has both negative and positive dimensions. The negative dimension of individual sovereignty connotes the power to exclude others from personal data, while the positive dimension includes the power to decide where your data goes and how it is to be used. They compare individual sovereignty to classical state sovereignty and posit that state sovereignty has both external and internal dimensions, in which the external dimension connotes no external inference, while the internal sovereignty means the state has the power to govern within its territory as it wills. Similarly, individual data sovereignty would mean the ability to exclude others from personal data, and the ability to operate within the informational self-determination sphere to pursue certain aims and goals with one’s data. In addition, in the broad context of sovereignty, they view power as the enabler of the exercise of sovereignty. In the context of individual data sovereignty, power means control over one’s individual data, that is, where it goes, who can access it, and what it is used for. Viewed from this perspective, individuals can exercise personal data sovereignty in its positive and negative dimensions (Hummel et al., 2019).

Further, Hummel et al. (2019) argue that individual data sovereignty can be facilitated in three ways: through consent, representation, and organisational level constraints. On consent, the authors state that informed consent would entail a balance between research participation and respect for the self-determination of the individual donor. In this respect they highlight mechanisms that allow for opting out of biobanks and withdrawing from research projects based on evolving preferences. In relation to representation, they point to representation of an individual donor’s will in the research governance processes to further their interests. Finally, on organisational-level constraints, they argue for supervisory oversight of institutions involved in data collection and processing through impartial licensing schemes and state legislation to ensure protection of the rights of individual donors.

Returning to Finland’s human genomic research framework, notably, the legal and policy documents expressly describe their objectives or goals as enabling individual control over their own genomic data. Illustratively, the Biobank Act sets out its objectives as promoting openness in the use of human biological samples and to ensure protection of privacy and self-determination in processing the samples (Ministry of Social Affairs and Health [Finland], 2013). Similarly, the Genome Act will establish the genome centre as a national reference database, and has, as one of its principles, the ability of individuals to control use of their genomic data (Tervo, 2021). The National Genome Strategy identifies the need for legislation to guarantee individual rights to control, manage and monitor own genomic data (Ministry of Social Affairs and Health [Finland], 2015).

The research framework also contains features of individual sovereignty in relation to control over use and management of their data. For instance, the Biobank Act provides for access to information for registered individuals. This allows them, upon request, to receive information on their samples such as whether their samples are stored in the biobank and the criteria, who can receive samples taken from them, who can access the samples, and information on transferring the samples from the biobank (Ministry of Social Affairs and Health [Finland], 2013).

In line with the three mechanisms to facilitate exercise of individual sovereignty discussed by Hummel et al. (2019) on consent, the Finnish research framework governing laws allow individuals to exercise control through issuing of consent to research participation. The Biobank Act’s provisions allow individuals to voluntarily consent to the use of their samples and data, to impose restrictions while issuing consent, and to cancel their consent at any time without any penalties (Ministry of Social Affairs and Health [Finland], 2013). In addition, the Act allows for change of consent or prohibition of the use of samples at any stage of the research process (Ministry of Social Affairs and Health [Finland], 2013). In this regard, it is arguable that individual data sovereignty is exercised, as the individual manages and controls how, for what, and by whom their samples are used.

In relation to organisational-level controls, the Finnish biobanks are regulated and monitored at the national level. First, prior to establishment, biobanks must obtain a positive statement from the National Committee on Medical Research Ethics confirming that the activities of the biobank comply with the protection of privacy and self-determination requirements (Ministry of Social Affairs and Health [Finland], 2013). Secondly, biobanks are supervised at the national level by Fimea to ensure that they maintain transparency in their biobanking activities (Ministry of Social Affairs and Health [Finland], 2023). Thirdly, biobanks are required to appoint a custodian whose duties are cast as obligations owed to the individual sample/data donor (Ministry of Social Affairs and Health [Finland], 2013). These organisational-level constraints enable individual data sovereignty as individuals retain control and management of how their samples and data are used.

### 4.3 A tale of two approaches to regulation of human genomic research: Mexico versus Finland

This section compares the above state approaches in the regulation of human genomic research. It is likely that there are more approaches and the choice of these two is random. Yet, both Mexico and Finland represent archetypes for two approaches to human genomic research that states have taken in the past decade in terms of the differences that characterise them and the seemingly unexpected similarities.

The general stance of Mexico’s approach may be described as follows. Human genomic resources are akin to other natural resources, and hence the need for the state to exercise a protective barrier to prevent foreign exploitation. The state’s role is thus viewed as to control and regulate access to and use of genomic resources. This position is actualised through establishment of the research institution INMEGEN, mapping of the national genome, and incorporation of the concept of genomic sovereignty in law.

Conversely, Finland’s approach is that genomic data can be used to improve the health outcomes of the Finnish people. The state’s role is thus to put in place the requisite framework for genomic data collection and utilisation. This position is actualised through the establishment of a legal framework on biobanks, mapping of the national genome, and establishment of the research framework through the National Genome Strategy which proposes a national genome centre and the genome law.

From the two approaches, this article identifies the following themes as the basis of comparison: philosophical underpinnings of the approaches; research infrastructure; and ownership of genomic data. As already demonstrated, Mexico and Finland have stark differences in their philosophical approaches. The Mexican approach is premised on genomic sovereignty, understood as state control of access to and use of genomic resources. This philosophy is rooted in the belief that genomic resources are national resources over which the state can exercise a protective barrier from foreign exploitation. In practice, this philosophy was embedded in the genomic sovereignty law which restricted export of human biological samples without state approval and the INMEGEN whose roles included surveillance of the samples. In the end, Mexico’s approach, which was underpinned by genomic sovereignty, proved impractical as it hindered international collaborations, hence violating the rights of both researchers and citizens generally. In addition, there was the inability of the state to exercise control due to the nature of population genomics, which defies territorial-based articulations of sovereignty. Finland’s research framework is premised on individual sovereignty, in which the individual donor of the samples and data exercises sovereignty by controlling and managing how their genomic data is used. The philosophy is rooted in human rights, in which individuals exercise informational self-determination. It is embedded in the legal framework on Biobanks and in the National Genome Strategy and its proposed national genome centre and genome, by vesting in individuals the power to control and manage use of their data.

On the research infrastructure, there are striking similarities. Both Mexico and Finland have established an institutional framework for genomic research, the INMEGEN in Mexico and the proposed national genome centre in Finland. In addition, both have laws to govern human genomic research: in Mexico, the genomic sovereignty law, while in Finland there is the Biobank Act and the proposed genome law. However, the point of departure is on the roles of the institutions and the objectives of the laws, which reflect the philosophy that underpins the overall state approach. In Mexico, the INMEGEN had the role of centralising and controlling genomic research. Similarly, the objective of the genomic sovereignty law was to restrict movement of samples without state approval. On the contrary, in Finland, the proposed national genome centre will facilitate international collaboration by setting up the national reference database of genomes with the necessary links to international databases and will provide centralised services for research projects and agreements. It will also plan and implement management of consents based on the individual right to decide. The genome law will enable individuals to control, manage and monitor the use of their genomic data.

Finally, how do the two state approaches deal with the issue of ownership, which in Finland is not deemed problematic, but which at the international level remains unsettled? In Finland, the general proposition is that genomic data are owned by the research institutions and biobanks. However, given that the research framework is underpinned by individual sovereignty, as discussed previously, it is reasonable to argue that the ownership is more nuanced and can perhaps be described as *custodianship*, given that significant control vests in individual donors. The Mexican proposition is the opposite. The issue of ownership of genomic resources undergirds the research framework. Genomic resources are viewed as national resources which can be subjected to exclusive state control. This proposition on ownership explains the violation of the rights of researchers and the complete disregard of the rights of individuals in Mexico’s research framework, which, in many states, would not pass constitutional scrutiny.

The foregoing discussion of the Mexican and Finnish approaches to human genomic research demonstrates that state approaches premised on genomic sovereignty are unworkable. First, this approach is not aligned to state obligations under international human rights law, specifically, the right to academic freedom for researchers, the right to privacy and self-determination for individuals, and the right to science. Secondly, the nature of genomic research defies state control premised on the idea of sovereignty, since sovereignty is territorial while polulations are fluid. In addition, the nature of genomic research is transnational, and hence obsession with national-level data is misplaced as national-level data on its own has limited utility value. Conclusively then, state approaches based on genomic sovereignty should be abandoned.

In the next section, the article explores obligations of the state under the right to science and in the specific context of human genomic research, in an effort to map out proposals on where state approaches should focus.

## 5 The right of everyone to enjoy the benefits of scientific progress and its applications in international human rights law

### 5.1 State obligations flowing from the right to science

The right of everyone to enjoy the benefits of scientific progress and its applications (the right to science) finds textual expression in the Universal Declaration of Human Rights (UN General Assembly, 1948) (UDHR) and in the International Covenant for Economic, Social and Cultural Rights (UN General Assembly, 1966) (ICESCR). Although not framed in identical wording, there are arguments in favour of construing the right as encompassing the right of everyone to access and contribute to knowledge and information and the right of everyone to benefit from scientific applications (Boggio and Romano, 2018; Yotova and Knoppers, 2020; Mancisidor, 2021). In addition, the right is formulated as part of cultural rights, and state practice has similarly evolved to treat the right as part of cultural rights (UN Human Rights Council, 2009). Arguments made in support of the right as a cultural right posit that both science and culture involve production of knowledge, innovation and creativity which support the full development of the person (Shaheed and Mazibrada, 2021).

Unlike other socio-economic rights, the right to science has not received much scholarly attention or in state implementation and has thus been said to be characterised by stunted development compared to other rights. Yotova and Knoppers (2020) citing Schabas refer to the right as the “sleeping beauty of human rights,” and Donders (2011), while discussing the reawakening of the right, refers to the right as recently “having its dust blown off”. Mancisidor (2021) notes that the textual positioning of the right at the end of both the UDHR and the ICESCR and its characterisation as a cultural right have contributed to its neglect. Even then, advances in science and technological innovation in the past decade have foregrounded the right. For instance, in the specific context of human genomic research there is scholarly work invoking the right in relation to genomic human research. Yotova and Knoppers (2020) have reviewed state practice on the right and argued that the right to benefit from science and its applications supports genomic data sharing. Elsewhere, Knoppers et al. (2014) anchor their proposal on an international code of conduct for sharing genomics and clinical data on the right to benefit from science and its applications. Below is a brief discussion on the right to science that maps out the state obligations as a prelude to the next section on state obligations in human genomic research.

A plain reading of the right to science as formulated in the ICESCR reveals positive obligations requiring states to “recognize the right of everyone to enjoy the benefits of scientific progress and its applications.” The UN Committee on Economic, Social and Cultural Rights (2020) elaborated on the elements of the right and the ensuing state obligations. It lists the elements of the right as: availability, accessibility, quality, and acceptability. *Availability* connotes a requirement of scientific progress and the protection and dissemination of scientific knowledge. Consequently, states have an obligation for conservation, development and diffusion of science by putting in place research infrastructure, funding research, promoting open science, and making accessible the findings and data of publicly funded research (UN Committee on Economic, Social and Cultural Rights, 2020). *Development* has been interpreted to mean state support for science while *diffusion* refers to equitable distribution of the benefits and applications of science, and *conservation* requires sustainable science that caters for present and future generations (Frick and Dang, 2021). *Accessibility* addresses the right of every person to access scientific progress and its applications without discrimination. States are thus to guarantee equal access to the applications of science to all, information on risks and benefits of science, and opportunity for all to participate in scientific progress (UN Committee on Economic, Social and Cultural Rights, 2020). The American Association for the Advancement of Science views accessibility as “a continuum of access” with the public on one end of the spectrum and scientists on the other (Frick and Dang, 2021). Quality relates to both creation of scientific knowledge and access to the benefits and applications of science. To this end, states are required to ensure ethical and responsible development of science and that only certified science is available to the general public (UN Committee on Economic, Social and Cultural Rights, 2020). An example of state failure to adhere to the element of quality in the application of science was the alleged United Arab Emirates use of faulty algorithms in diagnosing tuberculosis in its immigration procedures, which resulted in denial of work permits for migrants (Frick and Dang, 2021). *Acceptability* means respect for cultural diversity and pluralism in that the right to participate in science and enjoy benefits of science and its applications should be implemented in a manner that accords with specific cultural and social contexts. *Acceptability* also connotes ethical standards including respect for dignity, privacy and autonomy of individuals (UN Committee on Economic, Social and Cultural Rights, 2020). Respect for cultural diversity can be achieved by having community advisory boards in research projects and multidisciplinary and plural ethical review boards.

While the right to science is to be achieved progressively and subject to availability of resources, akin to other rights in the ICESCR, the right imposes obligations of a general nature on states which are of immediate implementation. To this end, the right to science requires states not to take retrogressive measures that impede enjoyment of the right. Such measures include imposition of policies that impede conservation, development and diffusion of science, the imposition of barriers to citizen participation, and adoption of laws and policies that prevent international collaborations (UN Committee on Economic, Social and Cultural Rights, 2020). In addition, states have an immediate obligation to eliminate discrimination in terms of participation in scientific progress and enjoyment of scientific benefits and its applications. Consequently, states should eradicate discrimination in the formulation and implementation of policies on the right to participate and benefit from science and its applications (UN Committee on Economic, Social and Cultural Rights, 2020).

The specific state obligations under the triptych typology are obligation to respect, to prevent or ensure, and to fulfil. Under the obligation to respect, states and their agencies should desist from interfering with the right to participate in and to enjoy the benefits of science and its applications. States therefore should not misinform the public on science and scientific research which could have the effect of eroding public trust in science, create obstacles for international collaboration among scientists, and arbitrarily limit internet access which could impede access to and the dissemination of scientific knowledge. The obligation to protect requires states to prevent violation of the right to science by non-state actors, including individuals and multinational corporations. The measures states should take to protect include: preventing non-state actors from applying discriminatory criteria in scientific research; ensuring and guaranteeing ethical standards for persons in scientific research; and protecting individuals in their familiar, social and cultural contexts when their right to science is violated. The UN Committee on Economic, Social and Cultural Rights (2020) recognises the dominance of private enterprises in the right to science and requires states to establish a legal framework that imposes a duty of human rights’ due diligence on multinational corporations. In addition, states have extraterritorial obligations to ensure that multinational corporations within their control do not violate the right to science when acting abroad. On the obligation to fulfil, states should put in place the requisite infrastructure—legal, institutional, financial and administrative—for the right to science. This includes: facilitating participation in international cooperation programmes, facilitating access to the internet, funding research and making scientific knowledge broadly available (UN Committee on Economic, Social and Cultural Rights, 2020).

Under Article 15(4) which refers to the gains of international cooperation in the right to science, states have an obligation to promote and facilitate scientific researchers to participate in the “international scientific and technical community” and to freely share data (UN Committee on Economic, Social and Cultural Rights, 2020). In addition, in recognition of the differences among states in science and technology, the ICESCR imposes special obligations on wealthier states to assist less wealthy states (UN Committee on Economic, Social and Cultural Rights, 1990).

### 5.2 State obligations in respect of human genomic research

Consistent with the idea of the interdependence and interconnectedness of human rights, this section examines state obligations in human genomic research from the prism of the right to science while reading in the right to health. The section draws from the approaches of Mexico and Finland in human genomic research for contextualisation.

The chronicles of the Mexican and Finnish approaches to human genomic research offer insights on its nature. It follows that: it is driven by large datasets that are tied to other data; it is characterised by transnational and public–private collaboration specifically that nationally bound genomic data has limited utility; it requires research infrastructure; and individual data and the inherent ethical issues are crucial. What then are the state obligations for the realisation of the right to science in this context?

At the outset, although ICESCR rights lend themselves to progressive implementation, as discussed above, states have immediate obligations. First, states are prohibited from taking retrogressive measures in relation to human genomic research. Retrogressive measures include removal of policies that are necessary to support scientific research and legal and policy changes that hinder international collaborations in science. Revisiting genomic sovereignty in Mexico, the result of this political and scientific policy agenda was that the law on genomic sovereignty hindered international collaboration as scientists could not share biological samples. While retrogressive measures may be permitted under the ICESCR, for instance in the case of natural disasters and severe recessions, they must be necessary and proportionate. As discussed, the philosophical underpinnings of genomic sovereignty are a postcolonial dispossession discourse which would not support a reading of it as a permissible measure. Mann et al. (2021) provide some direction on the evaluation of retrogressive measures under the ICESCR rights, such as a country’s level of development; economic recession; whether the country is involved in international conflict; and whether the country has sought assistance. Therefore, the concept of genomic sovereignty negates the right to science as it constitutes an unjustifiable retrogressive measure. The core state obligations require the state to take measures that enhance development, diffusion and the conservation of science. There are valuable lessons from Finland’s approach in which the state has facilitated research infrastructure, such as the national genome centre. This will create a national reference database for genomes with international links, thus allowing international collaborations in line with the obligation to support science.

The second set of state obligations that is immediate and not subject to progressive measures is non-discrimination. The formulation of the right to science uses the term *everyone*. Significantly, invocation of the term *everyone* in relation to participation in science means that not only researchers but also the public must participate in science and scientific progress without discrimination. States are thus directed to ensure that marginalised segments of the population can participate in genomic research and also enjoy the benefits and applications of scientific progress.

Flowing from non-discrimination in the right to science is the requirement of participation. On the framework for participation, Mann et al. (2021) point out two areas in which everyone can participate: in active dialogue between scientists and the public to create public trust and promote citizen science; and in data donation. The question then arises on state obligations in this regard. The element of accessibility in the right to science obligates states to put in place measures for everyone to participate in science. In addition, under the obligation to fulfil, the state should put in place legal, institutional and budgetary infrastructure that allows for participation of both citizens and scientists. Furthermore, in relation to data donation, states have an obligation to make data available through adoption of policies that encourage citizens to participate in research. Drawing from Finland’s approach, as discussed earlier, the philosophical underpinning of Finland’s genomic research is individual data sovereignty, which encourages data donation as citizens control and manage their data in genomic research. This proposition finds support in Finland’s legislation and policies, for instance the Biobank Act, the proposed Genome Act and the National Genome Strategy, which ensure that individuals control and manage their data.

Moreover, on transnational data sharing, the element of availability imposes a duty on states to support science. In relation to human genomic research, this implies sharing of data. Yotova and Knoppers (2020) also argue that the obligation of states to diffuse scientific knowledge would require states to share genomic data from a global public goods approach. As pointed out earlier, the Mexican concept of genomic sovereignty restricted transnational sharing of data of the Mexican genome and ultimately inhibited development and diffusion of science given that national genomic datasets have little utility value for genomic research.

On the centrality of individual and the inherent ethical issues, the key state obligations can be derived from the element of acceptability and the state’s specific obligation to protect. The element of acceptability requires states to ensure that scientific research incorporates ethical standards that respect privacy, autonomy and dignity of the individual, as well as minimisation of harm and maximisation of benefits. Relatedly, the obligation to protect requires states to prevent violation of rights by non-state actors through adoption of legislation and judicial remedies in instances of violation. To that extent, the approach taken by states in human genomic research should ensure that individual rights are respected and upheld. As demonstrated, the rights in issue are individual privacy and informational self-determination and human dignity. At the outset, it is also apparent that state approaches to human genomic research anchored on genomic sovereignty violate individual rights to human dignity by regarding individual personal data as a national resource and also violate privacy and informational self-determination by disregarding the rights of individuals in relation to their individual data. Taking lessons from Finland’s approach, the nature of human genomic research demands that human rights be at the core of the research infrastructure due to the centrality of the individual from the perspective of genomic data and application of the outcomes of scientific progress.

Finally, on the research infrastructure, the obligation to fulfil imposes a positive duty on states to actively facilitate the advancement of science. As noted, human genomic research is reliant on research infrastructure such as institutional frameworks for collecting and processing samples and data and the supporting legal and policy framework, including international linkages and collaborations. States therefore have an obligation to allocate funding for research infrastructure. A key lesson from the Mexican and Finnish approaches is the philosophical underpinning that anchors the research infrastructure. As demonstrated, philosophical underpinnings that view genomic resources as national resources are inclined to establish research infrastructure that hinders the right to science, as in the case of Mexico and genomic sovereignty.

## 6 Conclusion

This article sought to enquire whether genomic sovereignty is the appropriate governance framework for access to and use of genomic resources. Debate on the governance framework for the human genome remains unsettled 25 years after the adoption of the Universal Declaration on the Human Genome and Human Rights. While the Declaration appears to favour the common heritage of mankind governance framework, at the time of adoption a number of states indicated the need for further deliberations. Twenty-five years on, developments in this regard have remained stunted. However, in this time, state practice in human genomic research has evolved to embody divergent approaches, in some instances in response to the proposed international governance framework and in others motivated by the desire to participate in the bio-economy heralded by the Human Genome Project. This article analysed the divergent approaches by two states, Mexico and Finland. While not conclusively settling the issue of the most appropriate governance framework, the article brings out insights on which approach best supports human genomic research and which approach best enables a state to participate in the genomic knowledge economy. A key finding is that given the centrality of the individual in human genomic research, state-centred approaches—anchored on the notion of state appropriation of genomic resources—such as that embodied by genomic sovereignty, cannot withstand human rights scrutiny and should be abandoned.

Turning to the issue of human rights, the article argues that even in the absence of a conclusive position at the political level on the governance framework, states have legally binding obligations in human genomic research under international human rights law. The ICESCR guarantees everyone the right to science and imposes certain binding obligations on states. Significantly, the ICESCR enjoys near universal ratification and no state has placed a reservation on the right to science (UN Office of the High Commissioner for Human Rights, 2023). To this extent, state parties have binding obligations regardless of the absence of a conclusive international governance framework on human genomic research. The article once again demonstrates that state-centred approaches to human genomic research violate the rights of individuals and those of researchers, hence running foul of binding ICESCR obligations. In addition, the article maps the state obligations under the right to science—pointing to where state approaches to human genomic research should focus.

Returning to the broader and vexing question of the appropriate governance framework, individual personality rights, state sovereignty and the common heritage concepts have always seemed at odds in governance of human genomic research and it often appears as if one should trump the other. This article, for a start, points to the possibility of marrying these competing regulatory approaches. Undoubtedly, the state remains the primary site of accountability for protection of human rights. In that regard states have a role to play in governance of human genomic research by implementing the right to science and ensuring the protection of the other rights at play. For the individual and their personality rights, the Finnish approach demonstrates the possibility of individuals taking control in the use of their genomic data and samples.

The take-home lesson for African states is that genomic sovereignty has no utility value in human genomic research and thus should be abandoned as the philosophical basis for governing human genomic research, regardless of its superficial appeal in light of Africa’s history of colonisation and dispossession. Human rights offer a more promising approach. First, African states have binding obligations under the right to science—notwithstanding availability of resources. Also, taking into account the indivisibility of rights, states have obligations under the right to health to facilitate enjoyment of the highest attainable standard of health. As we have argued, African states should implement their human rights obligations in the context of human genomic research by putting in place the necessary research infrastructure, including ethical and legal frameworks to protect individual rights, and by facilitating international collaborations to foster research.

## Data Availability

The original contributions presented in the study are included in the article/Supplementary Material, and further inquiries can be directed to the corresponding author.
